# Temperature stable mid-infrared GaInAsSb/GaSb Vertical Cavity Surface Emitting Lasers (VCSELs)

**DOI:** 10.1038/srep19595

**Published:** 2016-01-19

**Authors:** A. B. Ikyo, I. P. Marko, K. Hild, A. R. Adams, S. Arafin, M.-C. Amann, S. J. Sweeney

**Affiliations:** 1 Advanced Technology Institute and Department of Physics, University of Surrey, Guildford, Surrey GU27XH, United Kingdom; 2 Walter Schottky Institut, Technische Universität München, Am Coulombwall 3, 85748 Garching, Germany

## Abstract

GaInAsSb/GaSb based quantum well vertical cavity surface emitting lasers (VCSELs) operating in mid-infrared spectral range between 2 and 3 micrometres are of great importance for low cost gas monitoring applications. This paper discusses the efficiency and temperature sensitivity of the VCSELs emitting at 2.6 μm and the processes that must be controlled to provide temperature stable operation. We show that non-radiative Auger recombination dominates the threshold current and limits the device performance at room temperature. Critically, we demonstrate that the combined influence of non-radiative recombination and gain peak – cavity mode de-tuning determines the overall temperature sensitivity of the VCSELs. The results show that improved temperature stable operation around room temperature can only be achieved with a larger gain peak – cavity mode de-tuning, offsetting the significant effect of increasing non-radiative recombination with increasing temperature, a physical effect which must be accounted for in mid-infrared VCSEL design.

There is growing interest in electrically pumped lasers that emit in the 2-3 µm wavelength region for applications such as pollution monitoring, medical diagnosis and chemical process control^1^. GaSb based type I quantum well edge-emitting lasers (EELs) are now well developed to provide room temperature CW operation^1^,^2^ in spite of being limited by non-radiative Auger recombination^1^,^3^ and inter-valence band absorption^4^. For example, CW power outputs of up to 500 mW have been recorded at 16 °C from 2.7 μm lasers with threshold current densities as low as 350 A/cm^2^ 2. The maximum wall-plug efficiency was 9.2% at a current of 2.4 A and a peak power of 2.5 W was recorded in pulsed-current mode operation at 20 °C. Characteristic temperatures of *T_0_ *= 71 K and *T_1_* = 86 K were measured for these devices^2^. EELs typically have highly divergent beams and with the required single-mode performance involve more costly fabrication and testing processes. Vertical Cavity Surface-Emitting Lasers (VCSELs), which emit in a direction perpendicular to the growth plane, are a preferred alternative for most applications^5^. The advantages of VCSELs include a single longitudinal mode (within the gain spectrum width), a high quality circular beam, low power consumption and ease of on-wafer testing^5^. Owing to the relatively small gain-length product of the active region in a VCSEL, high reflectivity Distributed Bragg Reflector (DBR) mirrors are required to accomplish sufficiently small mirror loss. The low effective cavity length, *L*, leads to a very large mode spacing, Δλ = λ^2^/2n_eff_*L*, where n_eff_ is the effective refractive index. Thus, in order to maximise the amount of gain available at the cavity mode, VCSELs are conventionally designed so that the gain peak and cavity mode align during device operation. The greater the energy splitting between the gain peak and the cavity mode, the higher the carrier density required to achieve the threshold gain. The VCSEL operating wavelength is determined by the cavity mode which has a weak temperature sensitivity as a result of the weak temperature dependence of the refractive index^6^. However, the gain peak wavelength has a much stronger temperature dependence owing to its dependence on the bandgap. The difference in temperature dependence between the cavity mode and gain peak can have a strong influence on the temperature sensitivity of the VCSEL characteristics. For optimum VCSEL performance the gain peak and the cavity mode alignment is engineered to obtain a minimum value of the threshold current (*I*_*th*_), and hence stable output power, around the operating temperature. However, due to the different temperature dependencies of the gain peak and cavity mode, there is an induced temperature dependent gain peak - cavity mode detuning^6^. This coupled with the intrinsic loss processes in low bandgap materials, such as Auger recombination and Inter-Valence Band Absorption (IVBA), are the main limitations for efficient performance of Sb-based mid-infrared VCSELs^7^. It is therefore important to understand the inter-play of these effects when developing and optimising VCSELs where non-radiative losses are present. It should be noted that combined effect of these factors depends strongly on the particular structure design and operating wavelength range. There are few, if any, studies in the literature discussing the optimization of mid-infrared VCSELs in this respect, which is the main subject of this work.

Here we report on the effects of detuning and non-radiative recombination in electrically pumped VCSELs with a room temperature emission wavelength of 2.6 μm. To study this we used novel high hydrostatic pressure and temperature tuning techniques. The VCSELs studied consisted of 7 compressively strained (1.7%) 10 nm thick GaInAsSb quantum wells grown within 8 nm thick GaSb barriers and 60 nm thick doped AlGaAsSb separate confinement layers. A high compressive strain is employed to increase the modal gain, reduce the threshold current density^8^,^9^, and increase the valence-band offset for an improved hole confinement^3^.The two DBRs consist of 24 bottom pairs of alternating lattice-matched AlAsSb/GaSb while the top DBR is made of four λ/4 layers of amorphous Si/SiO_2_. A buried tunnel junction (BTJ) of 12 μm diameter confines current to the active region, reduces the hetero-interface resistivity and is also useful for lateral index guiding^5^,^10^. Details of the structure and growth of the VCSEL have been reported elsewhere^5^,^10^. Similar 2.6 μm VCSELs demonstrated a single-mode operation up to a heat-sink temperature of 55 °C^10^. Devices with aperture diameters of 6 μm showed maximum output powers of 0.3 mW at room temperature with quantum efficiencies ~10% and threshold current density of 5 kA/cm^2^ 10.

To investigate the effects of non-radiative recombination in the VCSEL without the effects of cavity mode detuning we studied an EEL with a nominally identical QW design. Measuring the properties of the EEL provides information about the intrinsic carrier recombination processes occurring in the gain material^11^,^12^. The wafer was processed into Fabry-Perot devices with a cavity length of 700 μm and a ridge width of 120 μm and was measured as-cleaved. The temperature dependence of *I*_*th*_ in the EEL was measured using a nitrogen-cooled Oxford Instruments static gas exchange cryostat connected to an Oxford Instruments temperature controller that regulated the temperature from 80 K to 300 K^13^,^14^. The threshold current of the EEL was found to be temperature sensitive, with a *J*_*th*_ of ~15 ± 1 A/cm^2^ at 80 K increasing to ~700 ± 20 A/cm^2^ at 300 K as shown in Fig. 1. The characteristic temperature, *T*_*0*_, of the EEL was 37 K for 200 K<*T*<300 K and 85 K for 80 K<*T*<200 K. The measured *J*_*th*_ in these devices is higher and with a lower *T*_*0*_ than the 2.7 μm double QW devices reported in ref. 2 which we believe is due to the fact that a higher current density is required in our single QW devices. This is due to the logarithmic gain versus carrier density relationship for QW lasers which means that SQW devices operate at a lower differential gain point than MQW devices. This gives rise to a higher threshold carrier density and, due to the strong carrier density dependence of Auger recombination, causes a higher *J*_*th*_ and lower *T*_*0*_ for the SQW devices. The temperature variation of the slope efficiency of our devices gives rise to a characteristic temperature of 131 K in the temperature range of 200–300 K (for comparison, in reference 2 *T*_*1*_ was measured to be 86 K in the temperature range of 290–330 K). The slope efficiency represents the differential efficiency of conversion of injected electrons into photons above threshold whereby a finite *T*_*1*_ value is indicative of temperature dependent optical losses and/or temperature dependent current injection efficiency.

This high temperature sensitivity (low *T*_*0*_) is often an indicator of increasing non-radiative recombination or optical losses as the temperature increases. To estimate the contribution of non-radiative recombination to the threshold current, a temperature dependent spontaneous emission analysis was undertaken^11^,^14^. In this experiment, pure spontaneous emission emitted from a 100 μm window milled in the substrate of the EEL is collected. Such emission will not have undergone the effects of gain and loss along the laser cavity and, as such, is therefore a measure of the fundamental radiative recombination in the devices. The measured integrated spontaneous emission, *L*_*spon*_, is proportional to the radiative component of the injected current *I*_*rad*_. Therefore, by measuring *L*_*spon*_ at threshold, the contribution of the radiative component to *I*_*th*_ may be extracted at various temperatures. Further details of this technique are reported in refs 12 and 14. To estimate the maximum fraction of the radiative current we make the assumption that *I*_*rad*_ is equal to *I*_*th*_ at the lowest measured temperature, i.e. we assume negligible non-radiative recombination at the lowest temperature. Thus, from these measurements we were able to estimate a minimum value of the non-radiative current component. The results in Fig. 1 show that at least 97% of the threshold current in the EEL at room temperature is due to non-radiative recombination. Since this is determined by intrinsic material properties, the same contribution of non-radiative recombination is correspondingly expected in the VCSEL with the same active region at a given current density.

To identify the nature of the dominant non-radiative current path in the EELs, high hydrostatic pressure dependent measurements were carried out. High hydrostatic pressure reversibly varies the bandgap of a semiconductor material enabling study of carrier recombination and loss processes in devices owing to their different and very specific dependence on the bandgap. The lasers were mounted in a high pressure gas cell with a sapphire window to collect laser emission and enclosed in a closed cycle helium cryostat. The cell was connected to a helium gas compressor which was capable of generating gas pressures up to 1000 MPa^15^. The pressure dependencies of *I*_*th*_ in the EEL is presented in Fig. 2 at *T* = 200 K and *T* = 292 K. This shows that the threshold current decreases with increasing pressure at both temperatures. Since the band offsets are pressure independent, this rules-out carrier leakage as a dominant non-radiative loss, since this would be pressure insensitive. The decrease in threshold current with pressure indicates that the dominant non-radiative process reduces strongly as the pressure increases which is the signature of an Auger recombination process^13^,^15^. Furthermore, we note that *I*_*th*_ decreases more strongly at higher T owing to the larger Auger contribution. From this data, we may also conclude that *T*_*0*_ increases with increasing pressure. This interesting result shows that as the Auger recombination process decreases with increasing pressure (increasing bandgap), the device becomes less temperature sensitive, confirming that the Auger recombination process dominates both *I*_*th*_ and its temperature dependence, *T*_*0*_, under ambient conditions. Whilst it is clear that Auger recombination dominates the device behaviour, it is not possible to identify the specific Auger process from this data. However, we note that the composition of the QWs leads to the condition that the spin-orbit splitting energy (Δ_so_) is larger than the bandgap. Hence, the problematic CHSH Auger process is energetically forbidden^13^. The high strain in the QWs will also serve to reduce CHLH process and hence we predict that the hot electron producing CHCC process is likely to dominate^12^,^13^. Carrier leakage cannot be fully excluded at temperatures above room temperature as the valence band offset value of 48 meV is only slightly exceeds the thermal energy at room temperature (26 meV). Therefore, holes leakage by thermionic emission may contribute into increased temperature-sensitivity, leading to lower *T*_*0*_. However, this is of secondary importance to Auger recombination, as can be deduced from the pressure dependence data.

Utilising the tuning effect of high pressure on the bandgap of the 2.6 μm EEL, the wavelength dependence of the *T*_0_ has been evaluated as presented in Fig. 3. With increasing pressure, the bandgap increases, causing the laser emission wavelength to decrease. Under high pressure the 2.6 μm EEL is tuned to operate at a shorter wavelength of 2.15 μm and *T*_0_ values in the temperature range 200-293 K are observed to increase with decreasing wavelength. It is shown that by tuning the 2.6 μm device by pressure to emit at 2.3 μm *T*_0_ become similar to the as-grown device emitting at 2.3 μm (*T*_0_ = 53 ± 5 K) but with a reduced threshold current. This again shows that whatever is determining the temperature sensitivity in these devices is also strongly bandgap sensitive, decreasing with increasing bandgap. Auger recombination is consequently likely to be the controlling influence on both the threshold current and its temperature sensitivity in these devices.

To confirm that both EEL and VCSEL devices have the same as-grown active region we compared in Fig. 4 reflectivity and electroluminescence (EL) spectra from the VCSEL (for electroluminescence a reference VCSEL device without the top DBR mirror) with the lasing wavelengths of the EEL (2.544 μm) and VCSEL (2.573 μm). A broad EL peak around 2.2 μm most likely originates from the higher bandgap bottom mirror materials.

This confirms that the VCSEL wavelength is determined by the cavity mode (the dip in the reflectivity spectrum) and that the assumption that the EEL and the VCSEL have the same active region is justified. From this it was assumed that the lasing peak energy in an EEL is a good indicator of the gain peak energy, while the measured lasing peak energy of the VCSEL corresponds to the cavity mode. Thus, by plotting the EEL and VCSEL lasing energies versus temperature we can measure the extent to which the gain peak and cavity mode align in the VCSEL, as shown in Fig. 5.

From the linear fit of data in Fig. 5 one can extrapolate to the temperature at which the gain peak aligns with the cavity mode. The results show that the gain peak in the EEL decreases with increasing temperature at the rate of −0.28 meV/K while the cavity mode changes at a much smaller rate of −0.05 meV/K. From these data, the gain peak and cavity mode in the VCSEL are estimated to align at *T* ≈ 330 K at an energy of 0.481 eV. This yields a gain-cavity detuning of 54 meV at 80 K and 8 meV at 300 K (see Fig. 5). In the absence of temperature dependent loss processes one would conventionally expect the threshold current to be minimised at approximately 330 K owing to the fact that the gain is maximised at the cavity mode. However, this overlooks factors such as the temperature dependence of the peak gain and non-radiative recombination which, as we now go on to show, significantly influence the temperature at which the threshold current is at a minimum.

The temperature dependence of the VCSEL threshold current is shown in Fig. 6. The minimum threshold current occurs around 220 K. With reference to Fig. 1, we note that the threshold current of the edge emitting laser increases strongly over this temperature range due to Auger recombination. In the VCSEL, this causes the minimum threshold current temperature to occur at a much lower temperature (*T* ~ 220 K) than the gain – cavity mode alignment temperature (*T* ~ 330 K). At low temperature, owing to the large detuning between the gain peak and the cavity mode energy, coupled with the fact that the gain spectrum itself is narrower at low temperature, *I*_*th*_ increases sharply and is higher at 80K than at 300 K. For temperatures between 200 and 230 K, *I*_*th*_ is approximately temperature insensitive owing to the competition between improving gain-cavity alignment and increasing non-radiative Auger recombination. The influence of de-tuning of the gain cavity alignment is illustrated in the insets of Fig. 6. Similar, albeit less extreme behaviour has previously been observed in 1.3 μm GaAsSb/GaAs VCSELs^7^. This is consistent with Auger recombination being much stronger in the mid-infrared VCSELs.

Hydrostatic pressure measurements^16^ were used to further confirm the photon energy corresponding to the gain peak – cavity mode alignment. Similar to the temperature measurements, we used the pressure dependencies of the lasing peaks of the VCSEL and EEL, to probe the cavity-mode and gain peak, respectively, as shown in Fig. 7. By extrapolating the data the gain peak – cavity mode alignment was found to be at ~0.482 eV (see Fig. 7). This value is in excellent agreement with that determined from the temperature induced tuning presented in Fig. 5.

In conclusion, we have demonstrated that the optimisation of mid-infrared VCSELs must account for the strong effect of Auger recombination. Our results show that non-radiative Auger recombination accounts for up to 97% of the injected current at threshold in 2.6 μm edge-emitting lasers. We have shown that for the VCSEL the interplay between such strong Auger recombination in the active region coupled with gain – cavity mode de-tuning causes the threshold current minimum to shift by >100 °C from that expected due simply to conventional gain-cavity alignment considerations. For the devices considered, the region of temperature insensitive operation can be moved to room temperature by shifting the gain peak to higher energy by altering the composition and/or quantum well thickness. This would however, give rise to a higher absolute value of *I*_*th*_ which, however, could be partly compensated by a reduced Auger recombination of the higher bandgap gain material. This work shows that for effective mid-infrared VCSEL design the coupling between gain peak – cavity mode detuning and strong non-radiative Auger recombination is essential.

## Additional Information

**How to cite this article**: Ikyo, A. B. *et al*. Temperature stable mid-infrared GaInAsSb/GaSb Vertical Cavity Surface Emitting Lasers (VCSELs). *Sci. Rep.* **6**, 19595; doi: 10.1038/srep19595 (2016).

**Data Availability**: Details of the data and how to request access are available from the University of Surrey publications repository at http://epubs.surrey.ac.uk/809594/

## Figures and Tables

**Figure 1:**
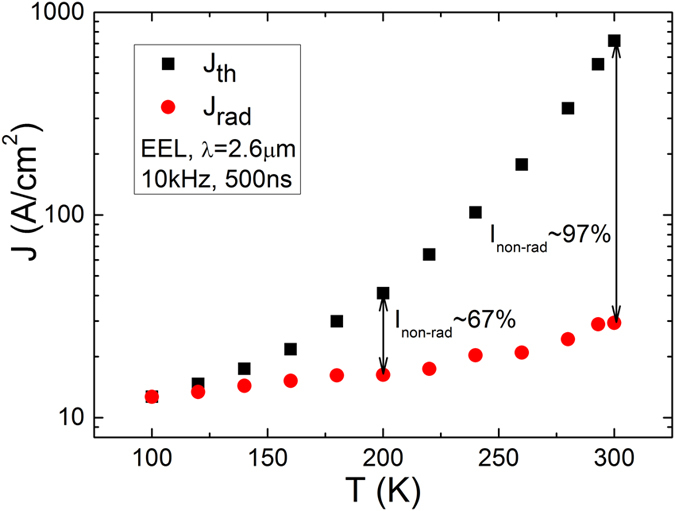
Temperature dependence of *J*_*th*_ (squares) and its radiative component *J*_*rad*_ in the EEL (circles). From this a relative contribution of current loss due to non-radiative processes can be estimated as shown for 200 K and 300 K assuming non-radiative current paths are negligible at the lowest T.

**Figure 2:**
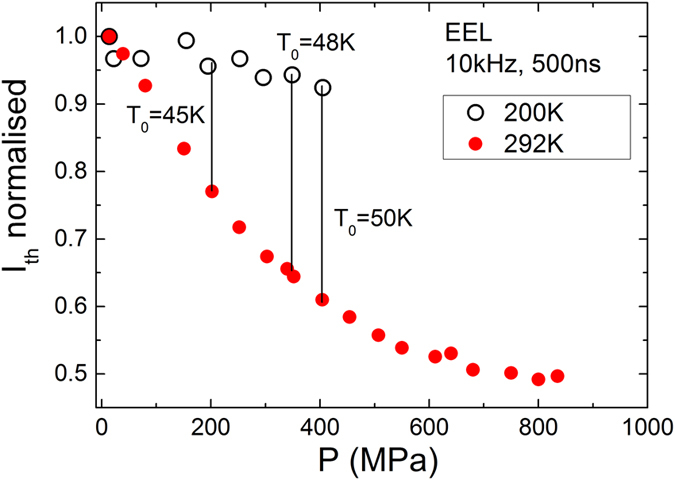
Pressure dependencies of threshold current in the EEL at 200 K and 292 K with calculated *T*_*0*_ values at different pressure.

**Figure 3:**
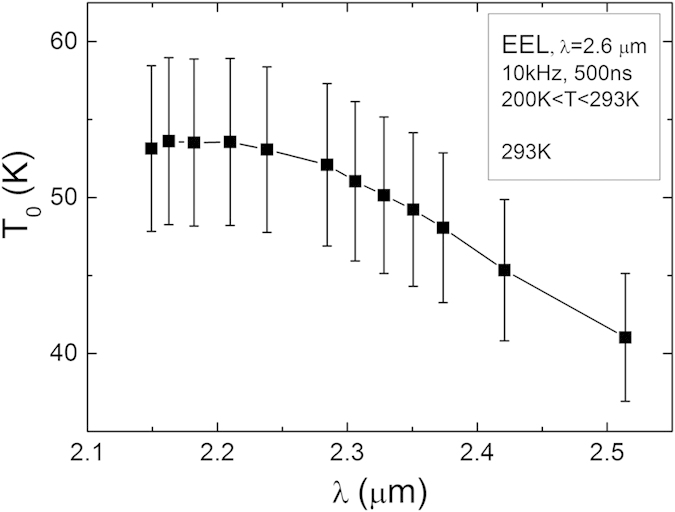
Wavelength dependence of *T*_0_ in the temperature range of 200–293 K. The lasing wavelength was tuned using high hydrostatic pressure.

**Figure 4:**
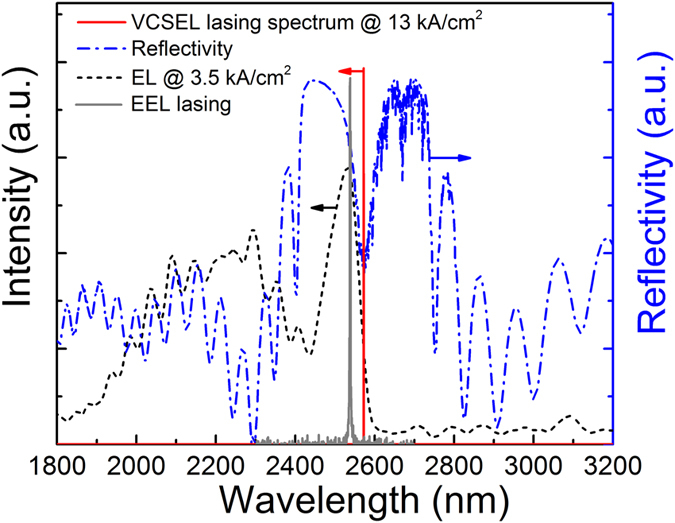
Reflectivity spectrum of the wafer from which the VCSEL was made and electroluminescence spectrum from a reference VCSEL device without the top mirror at current density of 3.5 kA/cm^2^. The grey curve is the RT lasing spectrum of the EEL (peak at 2.544 μm). A lasing spectrum from the VCSEL (peak at 2.573 μm) was measured at 13 kA/cm^2^.

**Figure 5:**
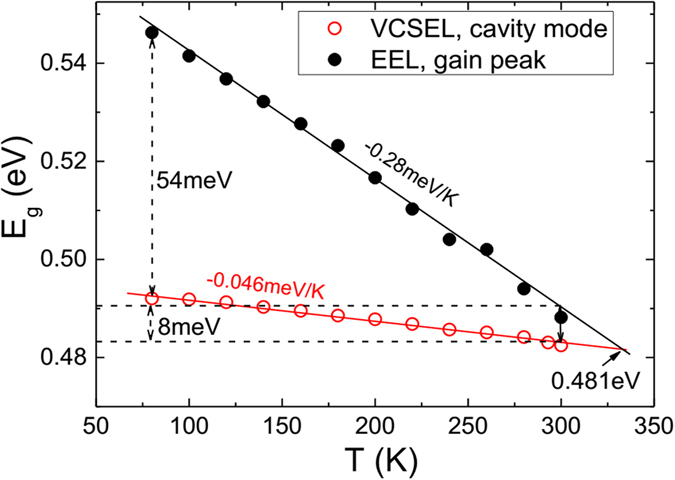
Temperature dependence of the lasing photon energy of the EEL (closed circles) represents the gain peak and temperature dependence of the lasing peak of VCSEL (open circles) represents the temperature variation of the cavity mode.

**Figure 6:**
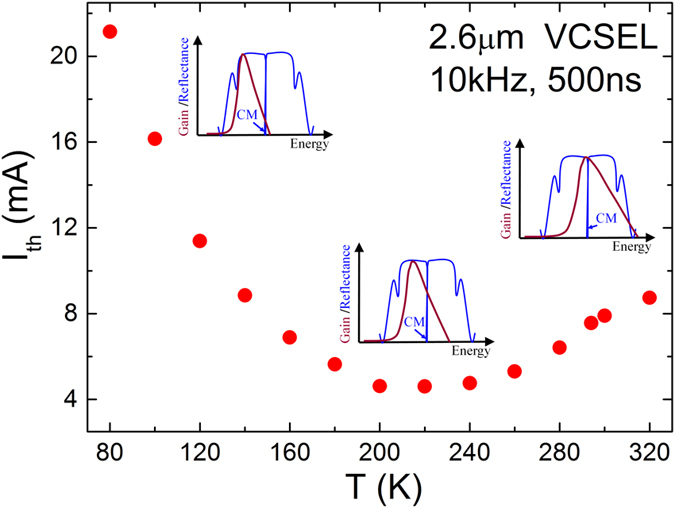
Temperature dependence of *I*_*th*_ in the VCSEL. Insets qualitatively illustrate the influence of detuning effect (see Fig. 4) in the measured temperature range.

**Figure 7:**
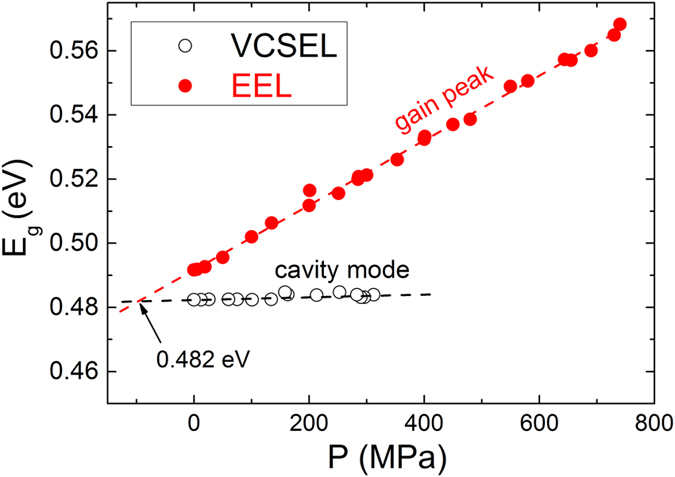
Pressure dependencies of the gain peak (lasing peak of the EEL) and of the cavity mode (lasing peak of the VCSEL). Photon energy corresponding to the gain peak – cavity mode alignment was found to be *E*_*g*_ ≈ 0.482 eV.
